# Recent Advances in the Development of Vaccines for Diabetes, Hypertension, and Atherosclerosis

**DOI:** 10.1155/2018/1638462

**Published:** 2018-09-24

**Authors:** Kongye Lu, Benli Su, Xiuxiang Meng

**Affiliations:** ^1^College of Laboratory Medicine, Dalian Medical University, Dalian, Liaoning 116044, China; ^2^Department of Clinical Endocrinology, The Second Affiliated Hospital of Dalian Medical University, Dalian, Liaoning 116027, China

## Abstract

Vaccines are commonly used in the prevention of infectious diseases. The basic principle of vaccination is to use specific antigens, endogenous or exogenous to stimulate immunity against the specific antigens or cells producing them. Autoantigen or oligo vaccination has been used for disease animal models. More recently humanized monoclonal antibodies have been successfully used for the treatment of neoplastic disorders or familial hypercholesterolemia. Humanized monoclonal antibody therapy needs repeated injection, and the therapy is expensive. Therapeutic vaccination can lead to persistent immunized or immune tolerant against the therapeutic molecule(s) or site. However, immunization against those endogenous substances may also elicit persistent autoimmune reaction or destruction that do harm to health. Therefore, rigorous studies are needed before any clinical application. In this review, we briefly reviewed vaccines used in protection against common metabolic diseases including atherosclerosis, hypertension, and diabetes mellitus.

## 1. Introduction

Over the past decades, the lifespan of a human being increased significantly; however, affluence and aging-related metabolic diseases (diabetes, hypertension, dyslipidemia, atherosclerosis, etc.) increased too. Metabolic disease usually results from the abnormality of normal chemical processes. With advances in understanding the mechanism of these metabolic disorders, great progress has been made in finding new drugs to correct the disease pathophysiology. As metabolic diseases are always associated with an unhealthy lifestyle or in some are associated with hereditary abnormalities, lifelong medication is needed and frequently results in low medication compliance. Therefore, scientists have screened the sea of molecular targets in trying to correct the pathophysiological process in a new way. More recently, trials of a humanized monoantibody, inhibiting proprotein convertase subtilisin/kexin type 9 (PCSK9) given 4 times over a period of one month showed a significantly long-term effect in decreasing low-density lipoprotein (LDL) cholesterol and a significant decrease in atherosclerotic events [1]. However, monoclonal antibodies are expensive and require repeated injection. Therefore, the replacement of monoclonal antibody therapy by vaccines might be an excellent alternative. Vaccine is a special biological preparation that elicits the adaptive immunity to defend against specific antigens. Although vaccine was originally designed to prevent or ameliorate infectious disease, it could also be used as a useful tool to provide a long-term antibody by eliciting adaptive immune responses. Recently, the vaccination of metabolic disease has made a great progress, especially in the treatment of dyslipidemia, atherosclerosis, diabetes mellitus, and hypertension.

### 1.1. Atherosclerosis

Atherosclerosis is classically defined as a chronic inflammation elicited by the accumulation of LDL particles over the intima in medium-sized and large arteries. Approximately, cardiovascular events occur every 43 seconds and cause one-third death in the United States, and cardiovascular disease (CVD) now is the first killer of women [2, 3]. Since the 1980s, the role lipid metabolism played in the atherosclerosis pathogenesis has been greatly elaborated. Researchers find atherosclerosis is not only merely an aggregation of LDL but also complex processes of chronic inflammation [4]. Both innate immunity and adapted immunity are evolved in this process. Although the details of the atherogenesis are still not fully understood currently, but some postulations consider oxidative stress as the major cause [5]. Once LDL is deposited and accumulated in the subendothelial space, it is converted to oxidized LDL (oxLDL) by reactive oxygen species generated from normal metabolism [6]. oxLDL is one of the initiators of the formation of fatty streaks, and it also accelerates the progress of atherosclerotic lesion by inducing the expression of chemokines, adhesion molecules, and the molecules involved including IL-1, TNF-*α*, C-C motif, and CCL2 [7]. In long term, oxLDL can lead to the apoptosis of endothelial and smooth muscle cells [8].

#### 1.1.1. Vaccine Target at CD99

CD99 is a leukocyte membrane protein that participates in the T cell activation, B cell aggregation, and monocyte transmigration [9]. Vaccines were developed by cloning the extracellular domain of murine CD99. When administrated orally, in the GI tract, genetic materials are transferred from a carrier to a host phagocyte. The phagocyte then expresses CD99 de novo in the cytosol and presents it on MHC molecules. By this approach, a CD99-specific and CD8-mediated cytotoxic response was successfully elicited. Atherosclerosis in aortic valve leaflets and carotid artery were reduced by 38% and 69%, respectively [10].

#### 1.1.2. Vaccine Target at VEGFR2

Vascular endothelial growth factor receptor 2 (VEGFR2) is expressed on the surface of the endothelial cells. Interacting with vascular endothelial growth factor (VEGF), VEGFR2 activates NF-*κ*B inside the endothelial cells [11]. Activated NF-*κ*B then leads to the expression of adhesion molecules like VCAM1, ICAM-1, and E-selectin, facilitating the adhesion of monocyte to endothelial cells [12]. DNA vaccine target at VEGFR2 was constructed by an approach the same as CD99 vaccination; VEGFR2 vaccination resulted in 4.6-fold increased cytolysis of VEGFR2-expressing cells by CD8+ T cells and protection against the initiation of atherosclerosis. In addition, those vaccines reduced the progression of preexisting advanced atherosclerotic lesions by 66% [13]. Phase I and phase II clinical trials using combined vaccines containing VEGFR2 against tumors have been conducted and shown a promising antiangiogenic effect [14, 15]. No clinical trial studies aimed at preventing atherosclerosis have been done yet.

#### 1.1.3. Vaccine Target at PCSK9

PCSK9 is another potential target. LDL-C interacts with LDL-R expressed on hepatocytes, and then LDL-C is endocytosed and degraded with LDL-R recycled to a cell surface. In this way, lipid level is reduced. However, PCSK9, a protein secreted by hepatic cells, is a negative regulator that inhibits the endocytosis of LDL-C and promotes the degradation of LDL-R. PCSK9 overexpression causes the upregulation of lipid level [16]. PCSK9-specific monoclonal antibodies including evolocumab (Amgen), bococizumab (Pfizer), and alirocumab (Aventis/Regeneron) have been approved to synergistically act with statins to lower LDL levels approximately by 60% [17]. PCSK9Q*β*-003 was found to be an ideal vaccine, showing an excellent performance [18]. AT04A vaccine was found to be another vaccine aimed at PSCK9 and exhibited a significant reduction of plasma lipids, systemic and vascular inflammation, and atherosclerotic lesions in the aorta in animal models [19].

#### 1.1.4. Vaccine Target at Apolipoprotein

It is widely acknowledged that LDL is a critical substance in the initiation and progression of atherosclerosis. Oxidized or small size dense LDLs lead to the activation of the intimal inflammation and formation of foam cell [5]. ApoB-100 is the major component of LDL; during the oxidation of LDL, it is degraded into numerous peptide fragments [20]. Approximately 102 peptides were found to be related to the immune responses in pooled human serum [21]. Nilsson's group is one of the most active on this field. They determined which epitopes are the products of the LDL oxidation [22]. Researchers have currently selected some effective candidates and developed corresponding vaccines. Among these candidates, p210 and p45 were found to be effective epitopes. Immunization with native p210 and p45 reduced atherosclerosis by 59% and 66%, respectively [23]. Vaccine aBp210, targeting at p210, induced 37% reduction in the development of atherosclerosis in immunized mice by activating T-regulatory cells (Tregs) [24]. Regions between amino acids 45–76 and 12–35 of apolipoprotein C-III were also found to be ideal sites. When tested in patients, atherosclerotic lesions are reduced by aiming at these sites [7].

#### 1.1.5. Vaccine Target at Heat Shock Proteins

Heat shock proteins (HSP) are promising candidates in antiatherosclerotic vaccine development [25]. Human HSP60 shows similarity with mycobacterial HSP65, and its atherogenic potential has been proven by both experimental and clinical studies [26, 27]. Under physical conditions, a human body is tolerant with HSP60; antibodies against HSP60 accelerate and perpetuate atherosclerosis [28]. An in silico analysis found that HSP60 vaccination might induce strong Th2 immune response in atherosclerosis [29]. HSP65-based vaccines reduced atherosclerosis and cholesterol levels with an increase in IL-10 level and decrease in IFN-*γ* level by intranasal immunization approach [30].

#### 1.1.6. Vaccine Target at *β*-2-Glycoprotein I

*Β*-2-Glycoprotein I (*β*-2-GPI) is a 50 kDa PLs-plasma glycoprotein which consists of five homologous complement control protein domains. Antiphospholipid antibodies (aPL) are the hallmark of antiphospholipid syndrome (APS) and St. Louis encephalitis (SLE). And anti-*β*-2-GPI antibody is one of aPL. Data suggests that the presence of anti-*β*-2-GPI is closely associated with a prothrombotic state. In APS and SLE patients, aPL contribute to oxidative stress and cause vascular damage through the activation of immune cells [31, 32]. Immunization of LDL-receptor-deficient mice with *β*-2-GPI resulted in the acceleration of fatty streak formation; the enhancement of the atherosclerotic lesions was further substantiated in an apoE murine model [33]. By inducing immune tolerance of *β*-2-GPI, early atherosclerotic lesion formation was reduced and was postulated mediated by regulatory T cells (Tregs) [34].

#### 1.1.7. Vaccine Target at CETP

Cholesteryl ester transfer protein (CETP) was first reported in 1978; it is a hydrophobic glycoprotein that promotes the transfer of cholesterol ester (CE) from HDL to LDL and VLDL in the exchange of triglycerides (TGs) [35]. An animal study found that the average size of atherosclerotic plaques in rabbits was reduced about 45% when treated with a chimeric vaccine AnsB-TTP-CETPC and the average thickness was decreased too [36]. Serum HDL was increased, and LDL was decreased in CETP-vaccinated rabbits [37]. However, a phase I human trial with CETi-1 did not significantly change CETP function and HDL level [38]. The CETP pathway as an antiatherosclerotic site was questioned. Clinical trials using agents that inhibit CETP activity resulted in increased mortality [39–41]. But recently, the result of a REVEAL trail contrasts with it; the study shows that inhibition of CETP by treating statin-treated patients with anacetrapib reduces the risk of having a coronary event [42].

### 1.2. Diabetes Mellitus

Diabetes mellitus is a group of chronic metabolic diseases characterized by chronic hyperglycemia. The common forms of diabetes are type 1 diabetes mellitus (T1DM) and type 2 diabetes mellitus (T2DM). T1DM developed due to profound *β* cell destruction by autoimmune attacks against pancreatic *β*-cell, whereas T2DM exhibits as an apparent insulin-resistant state with decompensated *β* cell function commonly due to an unhealthy lifestyle and overweight/obesity. In 2013, there are approximately 382 million patients suffering from diabetes; this number may increase up to 592 million by 2035 [43]. Autoimmune against pancreatic *β* cell involves autoimmune trigger(s), autoimmune establishment, inflammatory attack, destruction of *β* cell, *β* cell instinct regeneration, and perpetual destruction. Vaccines are designed for these processes to protect from trigger(s), to induce immunotolerance, to stop or ameliorate immune attack, and to promote *β* cell generation and tolerance to environmental or instinct insults. For T2DM, although a multiple genetic inheritance predisposition is established but is only accountable for about 15% for its development, environmental especially lifestyle factors account for 85% of its development. In between the affluence of food, lack of activity, and development type 2 diabetes, there are currently notified eleven pathological pathways [44], and *β* cell decompensation against insulin resistance is the key in diabetes development. Some are related to inflammatory enhancement against pancreatic *β* cell damage. Therefore, vaccines designed for the prevention or medication are related to these pathways.

#### 1.2.1. Vaccines against T1DM

T1DM is developed because of the elimination of *β* cells by cytotoxic T cells. This process is carried mostly by autoimmunity, which makes it possible to treat it with vaccines. Autoimmunity against pancreatic *β* cell involves autoimmune trigger(s), autoimmune establishment, inflammatory attack, destruction of *β* cell, *β* cell instinct regeneration, and perpetual destruction. Vaccines are designed for these processes to protect from trigger(s), to induce immunotolerance, to stop or ameliorate immune attack, and to promote *β* cell generation and tolerance to environmental or instinct insults.

Although the very reasons for T1DM remain unknown, epidemiological studies have shown that enterovirus infections were implicated, in particular, by Coxsackievirus B (CVB) serotypes [45]. The exact contribution of Coxsackievirus B (CVB) serotypes in the pathogenesis of T1DM remains elusive. Stone et al. [46] constructed a CVB1 vaccine and tested its efficacy. The result showed a 100% protection from virus-induced diabetes, no loss of any insulin-producing *β* cells, and no pancreas destruction.

T1DM is an autoimmune disorder in which *β* cells are under attack of a body's own T cells. Studies trying to control or alleviate this process have been carried for decades. GAD is a major target of the autoimmune response in T1DM. Randomized controlled clinical trials of a GAD+ alum vaccine in human participants revealed conflicting results so far [47–49]. A meta-analysis aimed at estimating the affectivity of GAD vaccines reported that there is 98% probability that 20 *μ*g GAD with alum administered twice yields a positive biological effect, but to reach clinically desirable reductions, the biological effect should be developed further [50]. Bacillus Calmette-Guerin (BCG) vaccine is another vaccine that may induce the production of TNF to eliminate autoreactive T cells and result in the remission of insulin production. In the phase I randomized control trials in 2001 and 2010, BCG vaccine successfully reversed T1DM [51, 52]. A newly reported 8-year-long clinical trial of BCG vaccine shows long-term and stable reductions in blood sugar and epigenetic changes in Treg signature genes for restored tolerance in humans with advanced T1DM [53]. In addition, phase II clinical trials testing the efficacy of BCG vaccine have been approved by FDA [54]. Dipeptidyl peptidase 4 (DPP4) also named as the CD26 lymphocyte marker was initially identified as a therapeutic target for T2DM [55]. But DPP4 inhibitors have shown many other benefits, for example, anti-inflammation [56]. And serum DPP4 activity increased in T1DM children [57]. These findings suggest that DPP4 may be used as a target for T1DM. Li et al. designed a vaccine, D41-IA2(5)-P2-1, which exhibited a significant control of hyperglycemia in NOD mice [58]. Another chimeric vaccine named as U-IA-2(5)-P2–1 (UIP-1) was designed by Li et al. and tried in mice. It successfully increased insulin level and reduced blood glucose level after immunization [59]. P277 is a peptide derived from HSP60, vaccines based on this peptide were under phase III clinical trials, and the existing studies show it is a well-tolerated and effective vaccine in T1DM [60].

#### 1.2.2. Vaccines against T2DM

The pathophysiology of type 2 diabetes mellitus remains unknown, but recent studies have strongly suggested obesity as a risk factor for T2DM [61]. According to the American Diabetes Association (ADA) “Standards of Medical Care in Diabetes,” obesity management can delay the progression from prediabetes to T2DM and may be beneficial in the treatment of T2DM [62]. Diet and physical exercise are the main ways to attain the obesity management; however, lifestyle manifestation fails to continue lifelong for some patients; therefore, many patients consider antiobesity vaccines as an alternative choice. There are mainly 4 targets for obesity vaccines now, including adipose tissue antigens, somatostatin, glucose-dependent insulinotropic polypeptide (GIP), and ghrelin [63]. Among these vaccines, only adipose tissue antigens were tested on human. Cytokine IL-1*β* is a key proinflammatory substance in the pathogenesis of T2DM. In KK-A(y) mice, a vaccine consisting of an IL-1*β* epitope peptide exhibited reduced weight gain, improved glucose tolerance and insulin sensitivity, and decreased *β* cell loss [64, 65]. In the further phase I/II clinical trials, vaccine Hil1bQb targeting IL-1*β* was found safe and well-tolerant [66]. DPP4 as mentioned above is an inhibitor of glucagon-like peptide-1 (GLP-1) glucose-dependent insulinotropic peptide (GLP). GLP-1 and GLP could regulate blood glucose level after a meal by stimulating insulin release, delaying gastrointestinal emptying, inducing satiety, decreasing glucagon release, and preserving *β* cell mass [67]. Therapeutic vaccine against DPP4 has shown efficacy and safety in glucose regulation in mice. Pang et al. designed another vaccine, D41-IP, aimed at DPP4. In a test in C57BL/6J mice, 15 min after glucose challenge, insulin level was significantly elevated, and 100% of mice survived compared to the control group [68]. No clinical trials about DPP4 vaccines have been done so far. Although the pathophysiology of T2DM is complex, in recent studies, the gut microbiome was considered to be related with many metabolic disorders including T2DM. The cytolysis of Gram-negative bacteria releases lipopolysaccharides (LPS) that induce proinflammatory cytokines and result in insulin resistance. It might be a possible target with further studies [69].

#### 1.2.3. Vaccines for Prevention of Infections and Diabetic Complications

Patients suffering from diabetes are much more likely to develop infections due to their deranged immune system. Increasing evidence suggests infections including pneumococcal infections, influenza infections, and hepatitis infections [70–72]. A number of scientific organizations like the ADA, World Health Organization (WHO), and United Kingdom Guidelines have well-defined guidelines for vaccination in diabetes. Resulting from hyperglycemia, diabetes patients are likely to suffer from diabetic complications in their elder ages. ATRQ*β*-001 is a vaccine motioned above now which was found functioning in the prevention of streptozotocin-induced diabetic nephropathy [73].

### 1.3. Hypertension

Hypertension is one of the chronic metabolic diseases. It may lead to severe consequences when failing to control blood pressure properly including stroke, heart failure, coronary heart, disease. Hypertension now is one of the most important risk factors of the onset of cardiovascular diseases [74]. However, the truth is the hypertension rate is on the rise in developing countries with no improvement in awareness or control rate when contrasted to developed countries. A systematic analysis of population-based studies from 135 populations from 968,419 adults in 90 countries reported a prevalence rate of hypertension in 2010 of 28.5% in high-income countries and 31.5% in low- and middle-income countries. Awareness, treatment, and control rate of hypertension were much lower in middle- and low-income countries than in high-income countries [75]. Another Prospective Urban Rural Epidemiology (PURE) study compared prevalence, awareness, treatment, and control of hypertension in urban and rural communities in high-, middle-, and low-income countries showing similar results. The treatment rates and control rates in China were 22% and 5.3%, respectively [76]. With the collaboration of health authorities, medical societies, and drug industry, situations might gain some improvements. But a more effective way is developing a radical treatment. Around six decades ago, researchers began experimenting with vaccines to control hypertension. Due to the irreplaceable role renin-angiotensin system (RAAS) played during hypertension development, most researches were based on studies against RAAS. The vaccine candidates against hypertension, namely, ATR12181, pHAV-4Ang IIs, CYT006-AngQb, AngI-R, and ATRQ*β*-001, have shown promising results. A vaccine, CYT006-AngQb, has passed the initial phase and moved into phase 2 trials [77].

#### 1.3.1. Renin

RAAS plays a vital part in the development of hypertension and blood pressure control. As an initiator of RAAS, renin plays an important part in hypertension. Since 1941, renin has been tested as a target to elicit immunity and to lower blood pressure [78]. However, early attempts to reduce blood pressure by vaccines against renin failed because of nephritis due to autoimmune issues [79]. Because renin is present in a substantial amount in the kidney, the development of renin vaccines was considered impossible during that time [80], whereas a new study tested six peptides derived from renin and reveals that antigenic peptide hR32 vaccine mimicking the ASP catalytic site of human renin shows low cross-reactivity and may be a novel target to develop renin vaccine [81]. But further clinical trials are required to confirm this finding.

#### 1.3.2. Vaccine Target at Angiotensin II and Its Receptors

Angiotensin-converting enzyme inhibitor (ACEI) and angiotensin II receptor blocker (ARB) are choices of antihypertensive agents, especially in patients with diabetes. Angiotensin II and its receptors are also ideal targets for vaccines. A study aimed at evaluating the efficiency and safety of angiotensin II vaccines in mice indicates that angiotensin II was a predictable target [82]. In animal models of hypertension, vaccine ATRQ*β*-001 against hypertension II receptor type 1 decreased blood pressure effectively through inhibiting angiotensin II function [83]. An angiotensin II receptor (AT1) vaccine ATR12181 attenuated the development of high blood pressure in animal models, and this vaccine was safe and was able to protect target organs from hypertensive damage [84]. During a multicenter, double-blind, randomized, placebo-controlled phase II clinical trial, immunization with CYT006-AngQb that targeted angiotensin II showed no severe adverse effect, which means it was safe and well tolerated. A 300 *μ*g dose reduced blood pressure in mild-to-moderate hypertensive patients during the daytime, especially in the early morning [85]. However, further studies are still required to estimate the long-term safety and effectivity. A novel DNA vaccine was constructed by plasmid carrying hepatitis B core-Ang II group; systolic blood pressure and mean blood pressure were successfully reduced in spontaneously hypertensive rats (SHRs) without T cell activation. In addition, perivascular fibrosis in the heart tissue was also significantly decreased [86].

#### 1.3.3. Vaccine Target at Angiotensin I

Angiotensin is formed by the action of renin on angiotensinogen, and it is further cleaved by angiotensin-converting enzyme (ACE) to form angiotensin II. There were two major carriers for angiotensin I (AI) reported in 2003; one was tetanus toxoid (TT), and the other one was keyhole limpet haemocyanin (KLH). In a two-dose clinical trial, KLH showed a suitable alteration to TT as a carrier protein for AI, and conjugated vaccine AI-KLH resulted in a significant immune response to AI [87]. A subsequent double-blind, placebo-controlled phase I/II clinical trial of angiotensin I vaccine PMD3117 demonstrated it was safe and effective in immunogenicity in human beings. However, this vaccine did not decrease blood pressure in clinical trials [88]. The main reason was a feedback between angiotensin II and rennin. By modifying angiotensin I, a novel peptide Ang-R was created; activity of angiotensin I was removed with immunogenicity retained. Ang-R exhibited a capability to induce an immune response against both angiotensins I and II, resulting in the decrease in blood pressure in spontaneously hypertensive rats (SHRs) [89].

## 2. Conclusion

Metabolic diseases are prevalent currently duo to maladaptation to modern food affluence and lifestyle, and their pathogenesis is complex. As the progress in the understanding of their pathogenesis, a sea of key substances has been found. Shortcomings of the current therapeutic paradigm were also notified, and a fresh new paradigm is needed. Vaccination might be once and for all a way of a therapeutic paradigm for metabolic diseases. Vaccines were designed, constructed, and assessed; some studies are promising. In addition, vaccines are much cheaper and more convenient than monoclonal antibodies. But there remain some critical problems. First, most of the vaccines were only tested on preclinical models and require further experiments. Second, the routes of administration varied a lot and affected the safety and stability of the vaccines. Third, adjuvants used in different vaccines influenced the results quite much; a desired adjuvant still needs further study. Lastly, but not the least, we all hope one administration could elicit immune response strong enough forever, but can these vaccines be reliable? If not, the schedule and durability can be a long and costly journey. No success is based on every problem well settled; the history of science is a tail of try. We shall deal with problems as we go forward. In conclusion, metabolic diseases are becoming the first noticeable disorder which threatens the health and longevity of human. Vaccines are powerful tools in this battle if used properly.

## References

[1] Blom D. J., Hala T., Bolognese M. (2014). A 52-week placebo-controlled trial of evolocumab in hyperlipidemia. *New England Journal of Medicine*.

[2] Cagle S. D., Cooperstein N. (2018). Coronary artery disease: diagnosis and management. *Primary Care: Clinics in Office Practice*.

[3] Reamy B. V., Williams P. M., Kuckel D. P. (2018). Prevention of cardiovascular disease. *Primary Care: Clinics in Office Practice*.

[4] Chrysohoou C., Kollia N., Tousoulis D. (2018). The link between depression and atherosclerosis through the pathways of inflammation and endothelium dysfunction. *Maturitas*.

[5] Taleb S. (2016). Inflammation in atherosclerosis. *Archives of Cardiovascular Diseases*.

[6] Suciu C. F., Prete M., Ruscitti P., Favoino E., Giacomelli R., Perosa F. (2018). Oxidized low density lipoproteins: the bridge between atherosclerosis and autoimmunity. Possible implications in accelerated atherosclerosis and for immune intervention in autoimmune rheumatic disorders. *Autoimmunity Reviews*.

[7] García-González V., Delgado-Coello B., Pérez-Torres A., Mas-Oliva J. (2015). Reality of a vaccine in the prevention and treatment of atherosclerosis. *Archives of Medical Research*.

[8] Gonzalez L., Trigatti B. L. (2017). Macrophage apoptosis and necrotic core development in atherosclerosis: a rapidly advancing field with clinical relevance to imaging and therapy. *Canadian Journal of Cardiology*.

[9] Moricoli D., Muller W. A., Carbonella D. C. (2014). Blocking monocyte transmigration in in vitro system by a human antibody scFv anti-CD99. Efficient large scale purification from periplasmic inclusion bodies in *E. coli* expression system. *Journal of Immunological Methods*.

[10] van Wanrooij E. J. A., de Vos P., Bixel M. G., Vestweber D., van Berkel T. J. C., Kuiper J. (2008). Vaccination against CD99 inhibits atherogenesis in low-density lipoprotein receptor-deficient mice. *Cardiovascular Research*.

[11] Celletti F. L., Waugh J. M., Amabile P. G., Brendolan A., Hilfiker P. R., Dake M. D. (2001). Vascular endothelial growth factor enhances atherosclerotic plaque progression. *Nature Medicine*.

[12] Kim I., Moon S. O., Hoon Kim S., Jin Kim H., Soon Koh Y., Young Koh G. (2001). Vascular endothelial growth factor expression of intercellular adhesion molecule 1 (ICAM-1), vascular cell adhesion molecule 1 (VCAM-1), and E-selectin through nuclear factor-*κ*B activation in endothelial cells. *Journal of Biological Chemistry*.

[13] Hauer A. D., van Puijvelde G. H. M., van Weel V. (2006). Vaccination against VEGFR2 attenuates initiation and progression of atherosclerosis. *Vascular Pharmacology*.

[14] Suzuki N., Hazama S., Iguchi H. (2017). Phase II clinical trial of peptide cocktail therapy for patients with advanced pancreatic cancer: VENUS-PC study. *Cancer Science*.

[15] Matsuyama M., Ishii H., Furuse J. (2015). Phase II trial of combination therapy of gemcitabine plus anti-angiogenic vaccination of elpamotide in patients with advanced or recurrent biliary tract cancer. *Investigational New Drugs*.

[16] Chackerian B., Remaley A. (2016). Vaccine strategies for lowering LDL by immunization against proprotein convertase subtilisin/kexin type 9. *Current Opinion in Lipidology*.

[17] Crossey E., Amar M. J. A., Sampson M. (2015). A cholesterol-lowering VLP vaccine that targets PCSK9. *Vaccine*.

[18] Pan Y., Zhou Y., Wu H. (2017). A therapeutic peptide vaccine against PCSK9. *Scientific Reports*.

[19] Landlinger C., Pouwer M. G., Juno C. (2017). The AT04A vaccine against proprotein convertase subtilisin/kexin type 9 reduces total cholesterol, vascular inflammation, and atherosclerosis in APOE^∗^3Leiden.CETP mice. *European Heart Journal*.

[20] Shah P. K., Chyu K. Y., Fredrikson G. N., Nilsson J. (2005). Immunomodulation of atherosclerosis with a vaccine. *Nature Clinical Practice Cardiovascular Medicine*.

[21] Reyes O. S., Chyu K. Y., Yano J. (2002). Immunization with a novel human apo B100 related peptide reduces atherosclerosis and inflammation in apo E null mice. *Journal of the American College of Cardiology*.

[22] Salazar-Gonzalez J. A., Rosales-Mendoza S. (2013). A perspective for atherosclerosis vaccination: is there a place for plant-based vaccines?. *Vaccine*.

[23] Fredrikson G. N., Björkbacka H., Söderberg I., Ljungcrantz I., Nilsson J. (2008). Treatment with apo B peptide vaccines inhibits atherosclerosis in human apo B-100 transgenic mice without inducing an increase in peptide-specific antibodies. *Journal of Internal Medicine*.

[24] Wigren M., Kolbus D., Dunér P. (2011). Evidence for a role of regulatory T cells in mediating the atheroprotective effect of apolipoprotein B peptide vaccine. *Journal of Internal Medicine*.

[25] Deniset J. F., Pierce G. N. (2015). Heat shock proteins: mediators of atherosclerotic development. *Current Drug Targets*.

[26] Kimura T., Tse K., Sette A., Ley K. (2015). Vaccination to modulate atherosclerosis. *Autoimmunity*.

[27] Wick G., Jakic B., Buszko M., Wick M. C., Grundtman C. (2004). The role of heat shock proteins in atherosclerosis. *Nature Reviews Cardiology*.

[28] Chyu K. Y., Dimayuga P. C., Shah P. K. (2017). Vaccine against arteriosclerosis: an update. *Therapeutic Advances in Vaccines and Immunotherapy*.

[29] Karkhah A., Saadi M., Nouri H. R. (2017). In silico analyses of heat shock protein 60 and calreticulin to designing a novel vaccine shifting immune response toward T helper 2 in atherosclerosis. *Computational Biology and Chemistry*.

[30] Long J., Lin J., Yang X. (2012). Nasal immunization with different forms of heat shock protein-65 reduced high-cholesterol-diet-driven rabbit atherosclerosis. *International Immunopharmacology*.

[31] López-Pedrera C., Barbarroja N., Jimenez-Gomez Y., Collantes-Estevez E., Aguirre M. A., Cuadrado M. J. (2016). Oxidative stress in the pathogenesis of atherothrombosis associated with anti-phospholipid syndrome and systemic lupus erythematosus: new therapeutic approaches. *Rheumatology*.

[32] Meroni P. L., Peyvandi F., Foco L. (2007). Anti-beta 2 glycoprotein I antibodies and the risk of myocardial infarction in young premenopausal women. *Journal of Thrombosis and Haemostasis*.

[33] George J., Afek A., Gilburd B. (1997). Atherosclerosis in LDL-receptor knockout mice is accelerated by immunization with anticardiolipin antibodies. *Lupus*.

[34] Benagiano M., Gerosa M., Romagnoli J. (2017). *β*2 glycoprotein I recognition drives Th1 inflammation in atherosclerotic plaques of patients with primary antiphospholipid syndrome. *Journal of Immunology*.

[35] Wang X., Li W., Hao L. (2018). The therapeutic potential of CETP inhibitors: a patent review. *Expert Opinion on Therapeutic Patents*.

[36] Gaofu Q., Jun L., Xiuyun Z., Wentao L., Jie W., Jingjing L. (2005). Antibody against cholesteryl ester transfer protein (CETP) elicited by a recombinant chimeric enzyme vaccine attenuated atherosclerosis in a rabbit model. *Life Sciences*.

[37] Ryan U. S., Rittershaus C. W. (2006). Vaccines for the prevention of cardiovascular disease. *Vascular Pharmacology*.

[38] Davidson M. H., Maki K., Umporowicz D., Wheeler A., Rittershaus C., Ryan U. (2003). The safety and immunogenicity of a CETP vaccine in healthy adults. *Atherosclerosis*.

[39] Barter P. J., Caulfield M., Eriksson M. (2007). Effects of torcetrapib in patients at high risk for coronary events. *The New England Journal of Medicine*.

[40] Schwartz G. G., Olsson A. G., Abt M. (2012). Effects of dalcetrapib in patients with a recent acute coronary syndrome. *The New England Journal of Medicine*.

[41] Lincoff A. M., Nicholls S. J., Riesmeyer J. S. (2017). Evacetrapib and cardiovascular outcomes in high-risk vascular disease. *The New England Journal of Medicine*.

[42] The HPS3/TIMI55–REVEAL Collaborative Group (2017). Effects of anacetrapib in patients with atherosclerotic vascular disease. *The New England Journal of Medicine*.

[43] Guariguata L., Whiting D. R., Hambleton I., Beagley J., Linnenkamp U., Shaw J. E. (2014). Global estimates of diabetes prevalence for 2013 and projections for 2035. *Diabetes Research and Clinical Practice*.

[44] Rea I. M., Gibson D. S., McGilligan V., McNerlan S. E., Alexander H. D., Ross O. A. (2018). Age and age-related diseases: role of inflammation triggers and cytokines. *Frontiers in Immunology*.

[45] Rewers M., Ludvigsson J. (2016). Environmental risk factors for type 1 diabetes. *The Lancet*.

[46] Stone V. M., Hankaniemi M. M., Svedin E. (2018). A Coxsackievirus B vaccine protects against virus-induced diabetes in an experimental mouse model of type 1 diabetes. *Diabetologia*.

[47] Ludvigsson J., Faresjö M., Hjorth M. (2008). GAD treatment and insulin secretion in recent-onset type 1 diabetes. *The New England Journal of Medicine*.

[48] Wherrett D. K., Bundy B., Becker D. J. (2011). Antigen-based therapy with glutamic acid decarboxylase (GAD) vaccine in patients with recent-onset type 1 diabetes: a randomised double-blind trial. *The Lancet*.

[49] Ludvigsson J., Krisky D., Casas R. (2012). GAD65 antigen therapy in recently diagnosed type 1 diabetes mellitus. *The New England Journal of Medicine*.

[50] Beam C. A., MacCallum C., Herold K. C. (2017). GAD vaccine reduces insulin loss in recently diagnosed type 1 diabetes: findings from a Bayesian meta-analysis. *Diabetologia*.

[51] Faustman D. L., Wang L., Okubo Y. (2012). Proof-of-concept, randomized, controlled clinical trial of Bacillus-Calmette-Guerin for treatment of long-term type 1 diabetes. *PLoS One*.

[52] Ryu S., Kodama S., Ryu K., Schoenfeld D. A., Faustman D. L. (2001). Reversal of established autoimmune diabetes by restoration of endogenous beta cell function. *The Journal of Clinical Investigation*.

[53] Kühtreiber W. M., Tran L., Kim T. (2018). Long-term reduction in hyperglycemia in advanced type 1 diabetes: the value of induced aerobic glycolysis with BCG vaccinations. *NPJ Vaccines*.

[54] Lilani Z., Ahmed A., Tazeem R., Naeem E. (2017). BCG vaccine an innovation for diabetes treatment in Pakistan?. *Journal of Ayub Medical College Abbottabad*.

[55] Pang Z. D. (2014). Development of a novel vaccine against dipeptidyl peptidase-4 in mice. *Circulation*.

[56] Yazbeck R., Howarth G. S., Abbott C. A. (2009). Dipeptidyl peptidase inhibitors, an emerging drug class for inflammatory disease?. *Trends in Pharmacological Sciences*.

[57] Iwabuchi A., Kamoda T., Saito M. (2013). Serum dipeptidyl peptidase 4 activity in children with type 1 diabetes mellitus. *Journal of Pediatric Endocrinology & Metabolism*.

[58] Li Y., Cao H., Li Y. (2017). Construction of a novel vaccine by conjugating a B-cell epitope of DPP4 to peptide IA2(5)-P2-1 to significantly control type 1 diabetes in NOD mice. *Vaccine*.

[59] Li Z., Fang J., Jiao R. (2017). A novel multi-epitope vaccine based on dipeptidyl peptidase 4 prevents streptozotocin-induced diabetes by producing anti-DPP4 antibody and immunomodulatory effect in C57BL/6J mice. *Biomedicine & Pharmacotherapy*.

[60] Ma Y. J., Lu Y., Hou J. (2011). Vaccination of non-obese diabetic mice with a fragment of peptide P277 attenuates insulin-dependent diabetes mellitus. *International Immunopharmacology*.

[61] Tron'ko N. D., Zak K. P. (2013). Obesity and diabetes mellitus. *Likars'ka Sprava*.

[62] American Diabetes Association (2018). 7. Obesity management for the treatment of type 2 diabetes: standards of medical care in diabetes-2018. *Diabetes Care*.

[63] Monteiro M. P. (2014). Obesity vaccines. *Human Vaccines & Immunotherapeutics*.

[64] Zhang Y., Yu X., Zha J., Mao L., Chai J., Liu R. (2018). Therapeutic vaccine against IL-1*β* improved glucose control in a mouse model of type 2 diabetes. *Life Sciences*.

[65] Zha J., Chi X. W., Yu X. L. (2016). Interleukin-1*β*-targeted vaccine improves glucose control and *β*-cell function in a diabetic KK-A^y^ mouse model. *PLoS One*.

[66] Cavelti-Weder C., Timper K., Seelig E. (2016). Development of an interleukin-1*β* vaccine in patients with type 2 diabetes. *Molecular Therapy*.

[67] Cornell S. (2012). Differentiating among incretin therapies: a multiple-target approach to type 2 diabetes. *Journal of Clinical Pharmacy and Therapeutics*.

[68] Pang Z., Nakagami H., Osako M. K. (2014). Therapeutic vaccine against DPP4 improves glucose metabolism in mice. *Proceedings of the National Academy of Sciences of the United States of America*.

[69] Witjes J. J., van Raalte D. H., Nieuwdorp M. (2015). About the gut microbiome as a pharmacological target in atherosclerosis. *European Journal of Pharmacology*.

[70] Yende S., van der Poll T., Lee M. (2010). The influence of pre-existing diabetes mellitus on the host immune response and outcome of pneumonia: analysis of two multicentre cohort studies. *Thorax*.

[71] Christenson B., Lundbergh P., Hedlund J., Örtqvist Å. (2001). Effects of a large-scale intervention with influenza and 23-valent pneumococcal vaccines in adults aged 65 years or older: a prospective study. *Lancet*.

[72] Reilly M. L., Poissant T., Vonderwahl C. W., Gerard K., Murphy T. V. (2011). *Incidence of acute hepatitis B among adults with and without diabetes, 2009-2010*.

[73] Ding D., du Y., Qiu Z. (2016). Vaccination against type 1 angiotensin receptor prevents streptozotocin-induced diabetic nephropathy. *Journal of Molecular Medicine*.

[74] Liu M., Li Y., Liu S., Wang W., Zhou M. (2014). Burden on blood-pressure-related diseases among the Chinese population, in 2010. *Zhonghua liu xing bing xue za zhi = Zhonghua liuxingbingxue zazhi*.

[75] Mills K. T., Bundy J. D., Kelly T. N. (2016). Global disparities of hypertension prevalence and control: a systematic analysis of population-based studies from 90 countries. *Circulation*.

[76] Chow C. K., Teo K. K., Rangarajan S. (2013). Prevalence, awareness, treatment, and control of hypertension in rural and urban communities in high-, middle-, and low-income countries. *JAMA*.

[77] Bairwa M., Pilania M., Gupta V., Yadav K. (2014). Hypertension vaccine may be a boon to millions in developing world. *Human Vaccines & Immunotherapeutics*.

[78] Wakerlin G. E., Johnson C. A., Gomberg B., Goldberg M. L. (1941). Reduction in the blood pressures of renal hypertensive dogs with hog renin. *Science*.

[79] Michel J. B., Galen F. X., Guettier C. (1989). Immunological approach to blockade of the renin-substrate reaction. *Journal of Hypertension*.

[80] Maurer P., Bachmann M. F. (2010). Immunization against angiotensins for the treatment of hypertension. *Clinical Immunology*.

[81] Qiu Z., Chen X., Zhou Y. (2013). Therapeutic vaccines against human and rat renin in spontaneously hypertensive rats. *PLoS One*.

[82] Nakagami F., Koriyama H., Nakagami H. (2013). Decrease in blood pressure and regression of cardiovascular complications by angiotensin II vaccine in mice. *PLoS One*.

[83] Chen X., Qiu Z., Yang S. (2013). Effectiveness and safety of a therapeutic vaccine against angiotensin II receptor type 1 in hypertensive animals. *Hypertension*.

[84] Zhu F., Liao Y. H., Li L. D. (2006). Target organ protection from a novel angiotensin II receptor (AT1) vaccine ATR12181 in spontaneously hypertensive rats. *Cellular & Molecular Immunology*.

[85] Tissot A. C., Maurer P., Nussberger J. (2008). Effect of immunisation against angiotensin II with CYT006-AngQb on ambulatory blood pressure: a double-blind, randomised, placebo-controlled phase IIa study. *The Lancet*.

[86] Koriyama H., Nakagami H., Nakagami F. (2015). Long-term reduction of high blood pressure by angiotensin II DNA vaccine in spontaneously hypertensive rats. *Hypertension*.

[87] Downham M. R., Auton T. R., Rosul A. (2003). Evaluation of two carrier protein-angiotensin I conjugate vaccines to assess their future potential to control high blood pressure (hypertension) in man. *British Journal of Clinical Pharmacology*.

[88] Brown M. J., Coltart J., Gunewardena K., Ritter J. M., Auton T. R., Glover J. F. (2004). Randomized double-blind placebo-controlled study of an angiotensin immunotherapeutic vaccine (PMD3117) in hypertensive subjects. *Clinical Science*.

[89] Hong F., Quan W. Y., Pandey R. (2011). A vaccine for hypertension based on peptide AngI-R: a pilot study. *International Journal of Cardiology*.

